# Supplementary material for: Pathogen‐specific B‐cell receptors drive chronic lymphocytic leukemia by light‐chain‐dependent cross‐reaction with autoantigens
Source: EMBO Mol Med. 2017 Sep 12;9(11):1482–90. doi: 10.15252/emmm.201707732 (PMC5666309; doi:10.15252/emmm.201707732)
Supplement: Supplementary file 1 — Appendix [file EMMM-9-1482-s001.pdf]

Pathogen-specific B cell receptors drive chronic lymphocytic leukemia by light chain-dependent cross-reaction with autoantigens

Nereida Jiménez de Oya, Marco De Giovanni, Jessica Fioravanti, Rudolf Übelhart, Pietro Di Lucia, Amleto Fiocchi, Stefano Iacovelli, Dimitar G. Efremov, Federico Caligaris-Cappio, Hassan Jumaa, Paolo Ghia, Luca G. Guidotti and Matteo Iannacone

Appendix

Appendix Table S1, page 1

FIGURE 1A

Two-way ANOVA (Bonferroni's multiple comparison test)	Summary	Adjusted P Value
**Week 20**		
VI10YEN x Eμ-TCL1 vs. Eμ-TCL1	*	0,0386
D_HLMP2A x Eμ-TCL1 vs. Eμ-TCL1	***	0,0001
**Week 24**		
VI10YEN x Eμ-TCL1 vs. Eμ-TCL1	***	< 0,0001
D_HLMP2A x Eμ-TCL1 vs. Eμ-TCL1	***	< 0,0001
**Week 28**		
KL25 x Eμ-TCL1 vs. Eμ-TCL1	***	< 0,0001
VI10YEN x Eμ-TCL1 vs. Eμ-TCL1	***	< 0,0001
D_HLMP2A x Eμ-TCL1 vs. Eμ-TCL1	***	< 0,0001
**Week 32**		
VI10YEN x Eμ-TCL1 vs. Eμ-TCL1	***	< 0,0001
D_HLMP2A x Eμ-TCL1 vs. Eμ-TCL1	***	< 0,0001
		< 0,0001
**Week 36**		
VI10YEN x Eμ-TCL1 vs. Eμ-TCL1	***	< 0,0001
D_HLMP2A x Eμ-TCL1 vs. Eμ-TCL1	***	< 0,0001

FIGURE 1B

Log-rank (Mantel-Cox) test	Summary	Adjusted P Value
Eμ-TCL1 vs VI10YEN x Eμ-TCL1	***	< 0,0001
Eμ-TCL1 vs D_HLMP2A x Eμ-TCL1	***	< 0,0001
Eμ-TCL1 vs WT	***	< 0,0001
VI10YEN x Eμ-TCL1 vs D_HLMP2A x Eμ-TCL1	**	0,004

FIGURE 1C

One-way ANOVA (Bonferroni's multiple comparison test)	Summary	Adjusted P Value
**SPLEEN**		
D_HLMP2A x Eμ-TCL1 vs. Eμ-TCL1	***	< 0,0001
D_HLMP2A x Eμ-TCL1 vs. KL25 x Eμ-TCL1	***	0,0009
VI10YEN x Eμ-TCL1 vs. WT	***	0,0007
Eμ-TCL1 vs. WT	***	< 0,0001
KL25 x Eμ-TCL1 vs. WT	***	< 0,0001
**INGUINAL LNs**		
Eμ-TCL1 vs. WT	*	0,0352
**LIVER**		
D_HLMP2A x Eμ-TCL1 vs. Eμ-TCL1	***	< 0,0001
D_HLMP2A x Eμ-TCL1 vs. KL25 x Eμ-TCL1	**	0,0017
VI10YEN x Eμ-TCL1 vs. WT	**	0,0025
Eμ-TCL1 vs. WT	***	< 0,0001
KL25 x Eμ-TCL1 vs. WT	***	< 0,0001
**PERITONEUM**		
D_HLMP2A x Eμ-TCL1 vs. Eμ-TCL1	***	< 0,0001
D_HLMP2A x Eμ-TCL1 vs. KL25 x Eμ-TCL1	***	< 0,0001
VI10YEN x Eμ-TCL1 vs. Eμ-TCL1	***	0,0001
VI10YEN x Eμ-TCL1 vs. KL25 x Eμ-TCL1	**	0,0066
Eμ-TCL1 vs. WT	***	< 0,0001
KL25 x Eμ-TCL1 vs. WT	***	< 0,0001

FIGURE 2A

Two-way ANOVA (Bonferroni's multiple comparison test)	Summary	Adjusted P Value
**Week 20**		
Eμ-TCL1 vs. VI10YEN x Eμ-TCL1	*	0,0384
**Week 24**		
Eμ-TCL1 vs. VI10YEN x Eμ-TCL1	***	< 0,0001
**Week 28**		
Eμ-TCL1 vs. VI10YEN x Eμ-TCL1	***	< 0,0001
**Week 32**		
Eμ-TCL1 vs. VI10YEN x Eμ-TCL1	***	< 0,0001
**Week 36**		
Eμ-TCL1 vs. VI10YEN x Eμ-TCL1	***	< 0,0001

FIGURE 2B

Log-rank (Mantel-Cox) test	Summary	Adjusted P Value
Eμ-TCL1 vs. VI10YEN x Eμ-TCL1	***	< 0,0001

FIGURE 2C

Two-way ANOVA (Bonferroni's multiple comparison test)	Summary	Adjusted P Value
**Week 28**		
Eμ-TCL1 vs. KL25 x Eμ-TCL1	***	< 0,0001

FIGURE EV1B

One-way ANOVA (Bonferroni's multiple comparison test)	Summary	Adjusted P Value
**PERITONEUM**		
Eμ-TCL1 vs. KL25 x Eμ-TCL1	**	0,0088
Eμ-TCL1 vs.VI10YEN x Eμ-TCL1	**	0,0036
Eμ-TCL1 vs.D_HLMP2A x Eμ-TCL1	**	0,0015

FIGURE EV1C

One-way ANOVA (Bonferroni's multiple comparison test)	Summary	Adjusted P Value
**LIVER**		
Eμ-TCL1 vs. WT	*	0,0322
Eμ-TCL1 vs. KL25 x Eμ-TCL1	***	0,0002
Eμ-TCL1 vs. VI10YEN x Eμ-TCL1	***	< 0,0001
Eμ-TCL1 vs. D_HLMP2A x Eμ-TCL1	***	0,0005

FIGURE EV2B

One-way ANOVA (Bonferroni's multiple comparison test)	Summary	Adjusted P Value
**SPLEEN**		
Eμ-TCL1 vs. VI10YEN x Eμ-TCL1	*	0,0192
**INGUINAL LNs**		
Eμ-TCL1 vs. VI10YEN x Eμ-TCL1	*	0,034
**LIVER**		
Eμ-TCL1 vs. VI10YEN x Eμ-TCL1	**	0,0092
**PERITONEUM**		
Eμ-TCL1 vs. VI10YEN x Eμ-TCL1	**	0,0073

FIGURE EV3

Two-way ANOVA (Bonferroni's multiple comparison test)	Summary	Adjusted P Value
**Eμ-TCL1 vs Eμ-TCL1+LCMV**		
**Week 20**	**	0,0028
**Week 24**	***	< 0,0001
**Week 28**	***	< 0,0001
**KL25 x Eμ-TCL1 vs KL25 x Eμ-TCL1+ LCMV**		
**Week 28**	***	< 0,0001
