# Supplementary material for: Pathogen‐specific B‐cell receptors drive chronic lymphocytic leukemia by light‐chain‐dependent cross‐reaction with autoantigens
Source: EMBO Mol Med. 2017 Sep 12;9(11):1482–90. doi: 10.15252/emmm.201707732 (PMC5666309; doi:10.15252/emmm.201707732)
Supplement: Supplementary file 2 — Expanded View Figures PDF [file EMMM-9-1482-s002.pdf]

Expanded View Figures

Figure EV1. Characterization of KL25 × Eμ-TCL1, VI10YEN × Eμ-TCL1, and D_HLMP2A × Eμ-TCL1 mice.

A TCL1 expression in the spleen of 8-week-old WT (gray), Eμ-TCL1 (black), KL25 × Eμ-TCL1 (red), VI10YEN × Eμ-TCL1 (blue), and D_HLMP2A × Eμ-TCL1 (green) male mice analyzed by RT–qPCR. *n* = 3. Data are representative of more than three independent experiments.

B Concentration of $CD5^+$ $CD19^+$ cells per ml of blood and peritoneal wash of 8-week-old WT (gray), Eμ-TCL1 (black), KL25 × Eμ-TCL1 (red), VI10YEN × Eμ-TCL1 (blue), and D_HLMP2A × Eμ-TCL1 (green) male mice. *n* = 6 (WT), 8 (Eμ-TCL1), 9 (KL25 × Eμ-TCL1), 8 (VI10YEN × Eμ-TCL1), and 9 (D_HLMP2A × Eμ-TCL1).

C Absolute numbers of $CD5^+$ $CD19^+$ cells in the indicated organs of the same mice described in (B).

D Representative flow cytometry plots showing transgenic BCR expression in B1a ($CD19^+$ $CD5^+$ $CD23^-$), B1b ($CD19^+$ $CD5^-$ $CD23^-$), and B2 ($CD19^+$ $CD5^-$ $CD23^+$) cells from 8-week-old WT (top panel) and KL25 × Eμ-TCL1 (bottom panel) male mice. Results are representative of more than 10 independent experiments.

E Representative flow cytometry plots showing transgenic BCR expression in B1a ($CD19^+$ $CD5^+$ $CD23^-$), B1b ($CD19^+$ $CD5^-$ $CD23^-$), and B2 ($CD19^+$ $CD5^-$ $CD23^+$) cells from 8-week-old WT (top panel) and VI10YEN × Eμ-TCL1 (bottom panel) male mice. Results are representative of more than 10 independent experiments.

F, G Representative flow cytometry plots showing proliferation (assessed by CFSE dilution, left panels) and activation (assessed by CD25 and CD69 upregulation, middle and right panels, respectively) of KL25 × Eμ-TCL1 (F) or VI10YEN × Eμ-TCL1 (G) B cells exposed or not to inactivated LCMV or VSV, respectively. Results are representative of three independent experiments.

Data information: Results are expressed as mean + SEM. *$P < 0.05$, **$P < 0.01$, ***$P < 0.001$. Exact *P*-values for each experiment are reported in Appendix Table S1. One-way ANOVA (Bonferroni's multiple comparison) was used in (A–C).
Source data are available online for this figure.

© 2017 The Authors

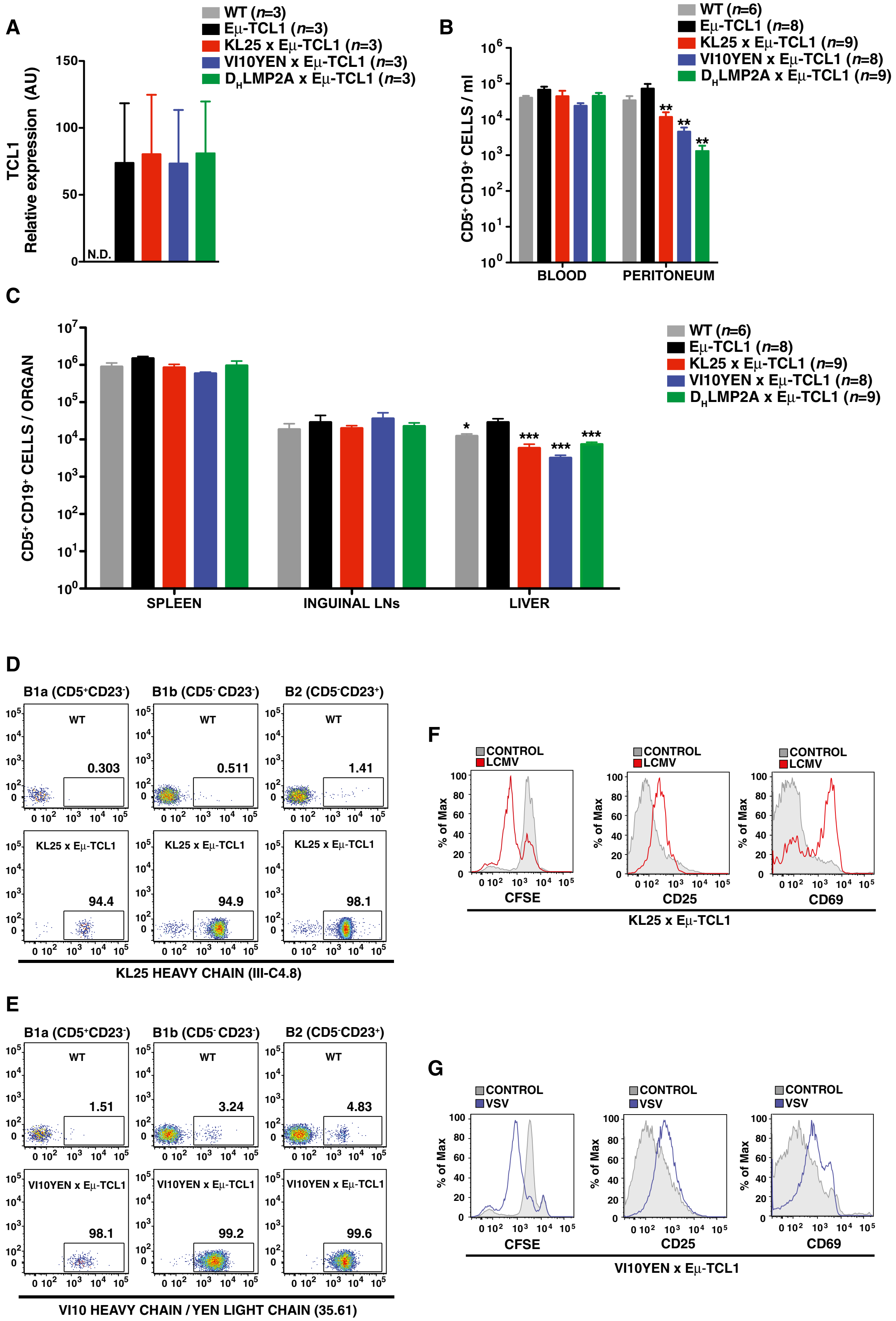
A
WT (n=3)
Eμ-TCL1 (n=3)
KL25 x Eμ-TCL1 (n=3)
VI10YEN x Eμ-TCL1 (n=3)
DHLMP2A x Eμ-TCL1 (n=3)
TCL1 Relative expression (AU)
N.D.
B
WT (n=6)
Eμ-TCL1 (n=8)
KL25 x Eμ-TCL1 (n=9)
VI10YEN x Eμ-TCL1 (n=8)
DHLMP2A x Eμ-TCL1 (n=9)
CD5+ CD19+ CELLS / ml
BLOOD
PERITONEUM
C
CD5+ CD19+ CELLS / ORGAN
SPLEEN
INGUINAL LNs
LIVER
D
B1a (CD5+CD23-)
B1b (CD5-CD23-)
B2 (CD5-CD23+)
WT
0.303
0.511
1.41
KL25 x Eμ-TCL1
94.4
94.9
98.1
KL25 HEAVY CHAIN (III-C4.8)
E
1.51
3.24
4.83
VI10YEN x Eμ-TCL1
98.1
99.2
99.6
VI10 HEAVY CHAIN / YEN LIGHT CHAIN (35.61)
F
CONTROL
LCMV
% of Max
CFSE
CD25
CD69
KL25 x Eμ-TCL1
G
CONTROL
VSV
VI10YEN x Eμ-TCL1

Figure EV1.

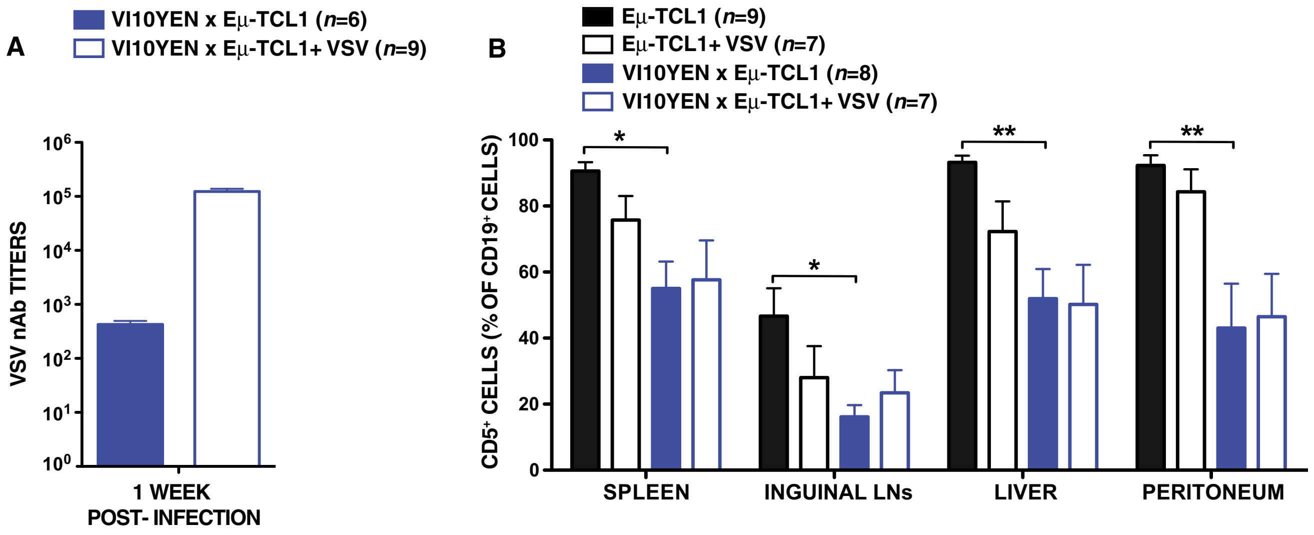

Figure EV2. VSV infection does not affect CLL development or progression.

A VSV neutralizing antibody (nAb) titers in the serum of VI10YEN × Eμ-TCL1 9-week-old male mice that were infected (open bars) or not (closed bars) with VSV 7 days earlier. $n = 6$ (VI10YEN × Eμ-TCL1) and 9 (VI10YEN × Eμ-TCL1 + VSV).

B Percentage of $CD5^+$ cells (out of total $CD19^+$ cells) in the indicated organs of Eμ-TCL1 (black) and VI10YEN × Eμ-TCL1 (blue) male mice that were infected (open bars) or not (closed bars) with 10^6 p.f.u. of VSV Indiana at 8 weeks of age and sacrificed 28 weeks later. $n = 9$ (Eμ-TCL1), 7 (Eμ-TCL1 + VSV), 8 (VI10YEN × Eμ-TCL1), 7 (VI10YEN × Eμ-TCL1 + VSV). One-way ANOVA (Bonferroni's multiple comparison).

Data information: Results are expressed as mean + SEM. $^*P < 0.05$, $^{**}P < 0.01$. Exact P-values for each experiment are reported in Appendix Table S1. Source data are available online for this figure.

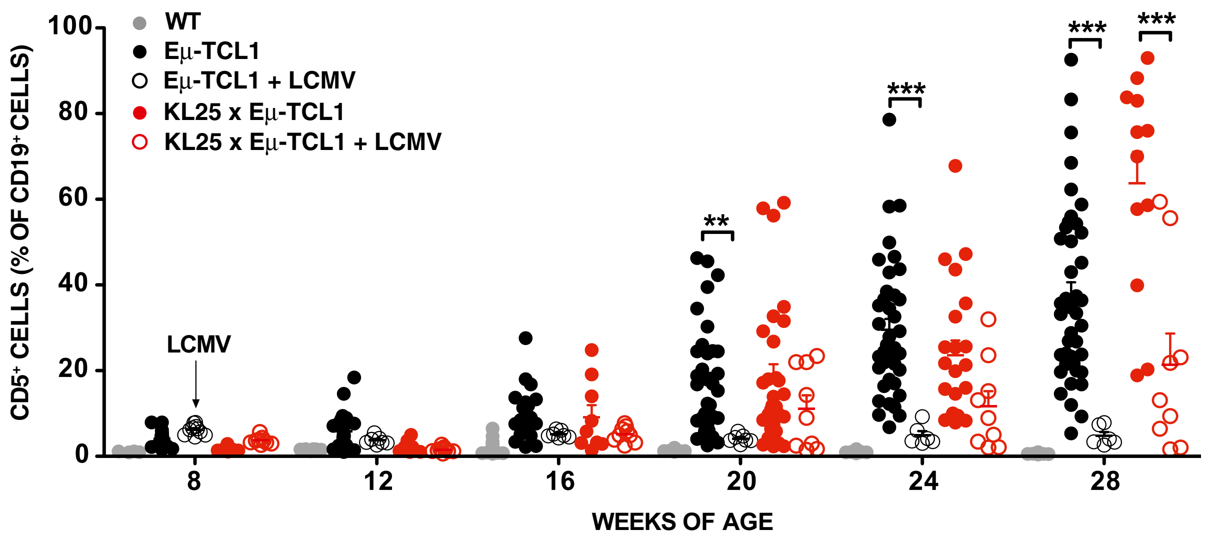

Figure EV3. LCMV infection prevents CLL development in Eμ-TCL1 mice.

Percentage of $CD5^+$ cells (out of total $CD19^+$ peripheral blood leukocytes) over time in WT (gray) and in Eμ-TCL1 (black) male mice that were infected (open symbols) or not (closed symbols) with LCMV. $n = 4–20$ (WT), 16–45 (Eμ-TCL1), 6–10 (Eμ-TCL1 + LCMV), 8–29 (KL25 × Eμ-TCL1), 9–11 (KL25 × Eμ-TCL1 + LCMV). Please note that the uninfected WT, Eμ-TCL1, and KL25 × Eμ-TCL1 controls are the same as the ones reported in Fig 1A. Results are expressed as mean + SEM. $^{**}P < 0.01$, $^{***}P < 0.001$. Exact P-values for each experiment are reported in Appendix Table S1. Two-way ANOVA (Bonferroni's multiple comparison) was used.

Source data are available online for this figure.

 © 2017 The Authors

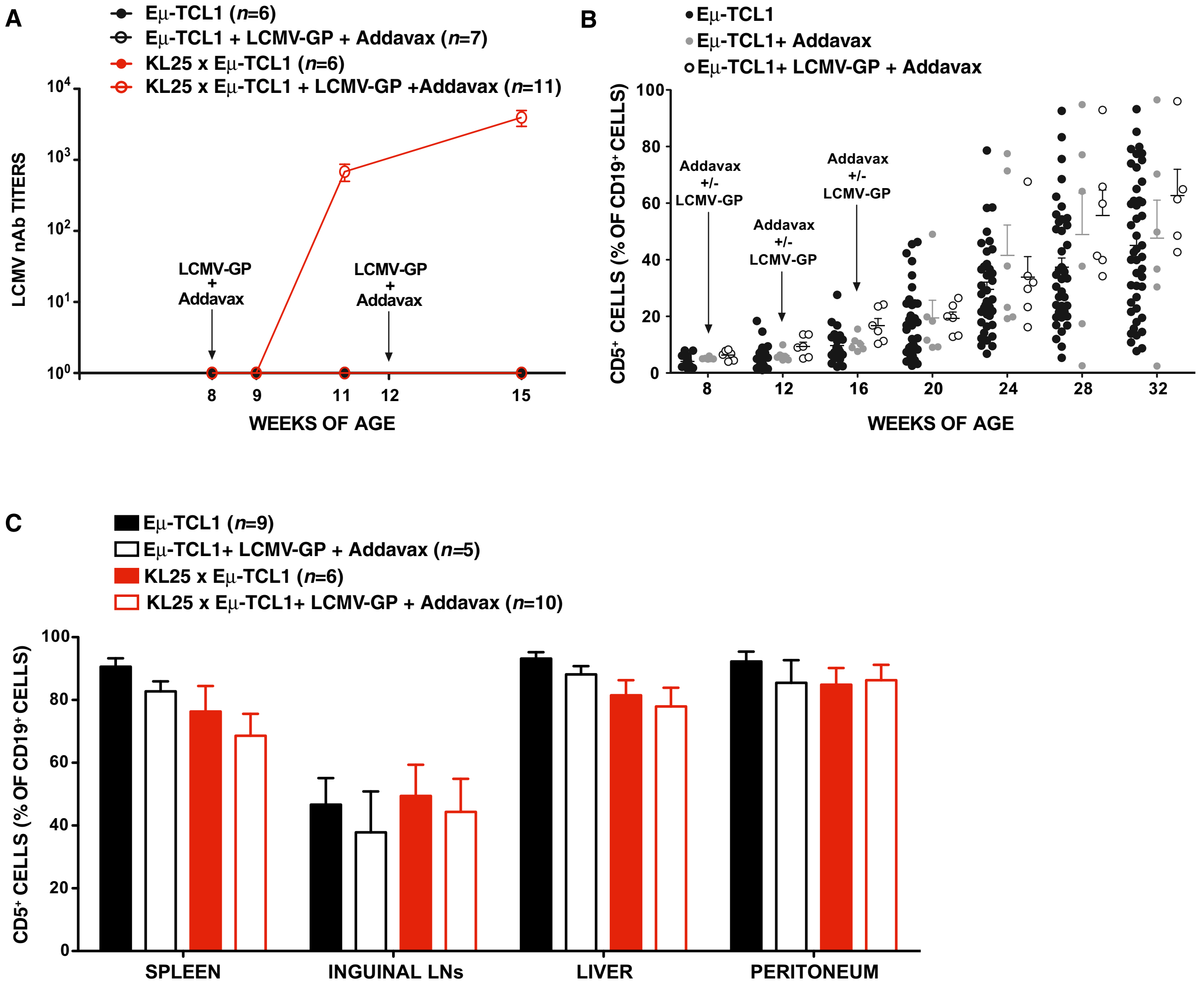

Figure EV4. Immunization with LCMV-GP does not affect CLL development or progression.

A LCMV neutralizing antibody (nAb) titers over time in the serum of Eμ-TCL1 (black) and KL25 × Eμ-TCL1 (red) male mice that were immunized (open symbols) or not (closed symbols) with LCMV-GP + Addavax. *n* = 6 (Eμ-TCL1), 7 (Eμ-TCL1 + LCMV-GP + Addavax), 6 (KL25 × Eμ-TCL1), 11 (KL25 × Eμ-TCL1 + LCMV-GP + Addavax).

B Percentage of $CD5^+$ cells (out of total $CD19^+$ peripheral blood leukocytes) over time in control Eμ-TCL1 mice (black symbols) or in mice that were injected intramuscularly with Addavax (gray) or with LCMV-GP + Addavax (open symbols).

C Percentage of $CD5^+$ cells (out of total $CD19^+$ cells) in the indicated organs of Eμ-TCL1 (black) and KL25 × Eμ-TCL1 (red) mice that were immunized (open bars) or not (closed bars) with LCMV-GP + Addavax and sacrificed 24 weeks after the first immunization. *n* = 9 (Eμ-TCL1), 5 (Eμ-TCL1 + LCMV-GP + Addavax), 6 (KL25 × Eμ-TCL1), 10 (KL25 × Eμ-TCL1 + LCMV-GP + Addavax).

Data information: Results are expressed as mean + SEM.
Source data are available online for this figure.

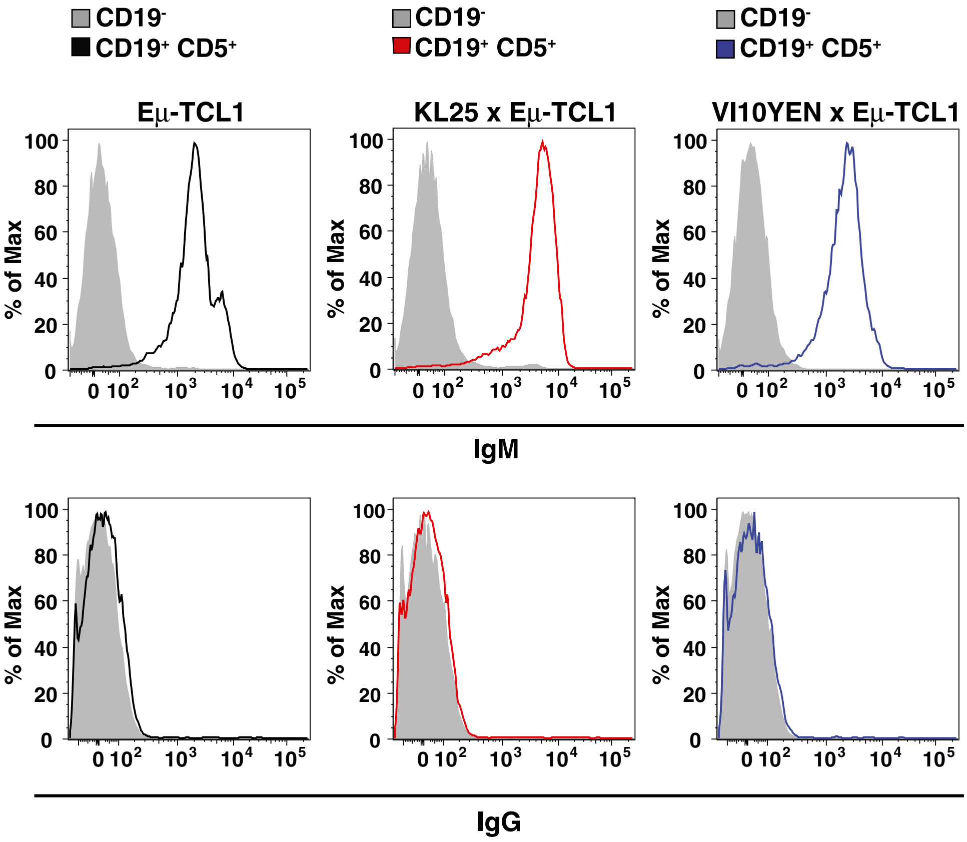

Figure EV5. CLL clones in Eμ-TCL1, KL25 × Eμ-TCL1, and VI10YEN × Eμ-TCL1 mice express IgM.

Representative flow cytometry plots of IgM (top panels) and IgG (bottom panels) expressions in $CD19^{-}$ cells (gray) and $CD19^{+}$ $CD5^{+}$ CLL cells (blue) in peripheral blood leukocytes isolated from 36-week-old Eμ-TCL1 (left panels), KL25 × Eμ-TCL1 (middle panels), and VI10YEN × Eμ-TCL1 (right panels) male mice.

 © 2017 The Authors
