# Supplementary material for: Pathogen‐specific B‐cell receptors drive chronic lymphocytic leukemia by light‐chain‐dependent cross‐reaction with autoantigens
Source: EMBO Mol Med. 2017 Sep 12;9(11):1482–90. doi: 10.15252/emmm.201707732 (PMC5666309; doi:10.15252/emmm.201707732)
Supplement: Supplementary file 6 — Source Data for Expanded View [file EMMM-9-1482-s013.zip › EMM_07322_EV_SD/FigEV1/EMM_07322_FigEV1A_SD.pdf]

FIG EV1A

	WT	Eμ-TCL1	KL25 x Eμ-TCL1	VI10YEN x Eμ-TCL1	DHLMP2A x Eμ-TCL1
Arbitrary units	0	41.36	42.52	37.27	154.34
	0.01	18.25	29.65	29.65	66.26
	0	162.02	168.9	153.28	22.16
