# Supplementary material for: Pathogen‐specific B‐cell receptors drive chronic lymphocytic leukemia by light‐chain‐dependent cross‐reaction with autoantigens
Source: EMBO Mol Med. 2017 Sep 12;9(11):1482–90. doi: 10.15252/emmm.201707732 (PMC5666309; doi:10.15252/emmm.201707732)
Supplement: Supplementary file 6 — Source Data for Expanded View [file EMMM-9-1482-s013.zip › EMM_07322_EV_SD/FigEV1/EMM_07322_FigEV1B_SD.pdf]

FIG EV1B

ORGANS	WT					
BLOOD	45175.39	49303.59	18714.82	36267.34	52134.25	42360.72
PERITONEUM	8093.248	41645.8	5725.515	76541.41	28623.09	44985.09

ORGANS	Eμ-TCL1								
BLOOD	127906.4	124278.5	88977.2	69504.76	32664.93	18910.18	38819.46	40122.43	
PERITONEUM	214098.7	65741.02	40398.79	62774.36	12951.54	16332.28	152100	19153.81	

ORGANS	KL25 x Eμ-TCL1								
BLOOD	45119.55	37657.46	11231.02	8487.271	92806.15	6468.147	16443.18	174702.2	9012.045
PERITONEUM	7343.209	8260.769	5164.576	3749.744	20600.56	1723.183	14031.51	40758.47	4275.492

ORGANS	VI10YEN x Eμ-TCL1								
BLOOD	14687.84	53403.61	21842.21	16283.79	20028.12	14631.5	23290.43	28645.43	
PERITONEUM	1626.162	4172.952	6569.341	2423.571	1054.947	405.9196	11018.24	9254.24	

ORGANS	DHLMP2A x Eμ-TCL1								
BLOOD	111909.5	39804.14	25543.67	22794.7	58946.09	53907.64	48648.04	30457.62	17670.46
PERITONEUM	824.4926	870.4017	5521.843	1593.764	1078.862	229.949	1141.596	92.91409	316.9446
