# Supplementary material for: Pathogen‐specific B‐cell receptors drive chronic lymphocytic leukemia by light‐chain‐dependent cross‐reaction with autoantigens
Source: EMBO Mol Med. 2017 Sep 12;9(11):1482–90. doi: 10.15252/emmm.201707732 (PMC5666309; doi:10.15252/emmm.201707732)
Supplement: Supplementary file 6 — Source Data for Expanded View [file EMMM-9-1482-s013.zip › EMM_07322_EV_SD/FigEV1/EMM_07322_FigEV1C_SD.pdf]

FIG EV1C

ORGANS	WT					
LIVER	18935.53	14463.36	11031.37	7167.085	12590.86	10174.06
INGUINAL LNs	17606.42	55766.96	14978.92	372.4885	12622.71	10335.61
MESENTERIC	172551.8	3724.017	18414.92	9005.574		
SPLEEN	606741.8	755432.6	1316455	1824510	495782.1	378524.2

ORGANS	Eμ-TCL1							
LIVER	44033.64	45182.07	48229.77	52247.79	7536.704	14699.06	12720	5764	
INGUINAL LNs	25587.42	135976.6	11982.44	17071.92	17646.93	16651.2	2184	2840.04	
MESENTERIC	151851.1	274599.8	31052.96	44670.54	13631.72	2608.64			
SPLEEN	1226065	1857916	2237409	2053588	1512981	954633.1	1459920	608256	

ORGANS	KL25 x Eμ-TCL1								
LIVER	8068.851	2441.66	4625.233	17667.7	4732.881	1198.101	4440.55	5256.829	4292.568
INGUINAL LNs	29967.48	41556.43	13455.14	12650.12	20558.3	18618.36	20612.17	15328.78	6659.264
MESENTERIC	99378.84	140778.5	38882.68	20946.82	13428.03	25396.03	9619.27	13301.94	8055.512
SPLEEN	1277090	789302.1	882018.8	343957.8	954551.8	155474.7	783685.3	2050481	379486.2

ORGANS	VI10YEN x Eμ-TCL1								
LIVER	2008.049	3536.804	4707.788	5654.999	897.213	1615.146	3243.168		3863.531
INGUINAL LNs	24466.18	90311.45	120263.3	5901.199	7513.826	20261.34	13997.48		7895.649
MESENTERIC	175316.3	114752.1	7832.466	17369.41	9893.48	23923.05	32068.15		2284.39
SPLEEN	637233.3	480747.3	812352.1	623191.1	796753.3	411265.6	402692		532437.5

ORGANS	DHLMP2A x Eμ-TCL1								
LIVER	5615.116	5562.432	13142.1	11083.1	9434.309	6297.72	6321.496	4416.642	4494.811
INGUINAL LNs	22365.71	45834.87	9022.815	458.7295	44960.99	23605.22	24307.26	13169.12	21097.63
MESENTERIC	119895	108432.6	50152.12	110402.5	17602.19	48787.43	63474.59	25189.89	18804
SPLEEN	686431.3	555303.4	210243.5	353241.3	3391541	768240.9	1210961	850853.3	559656.9
