# Supplementary material for: Pathogen‐specific B‐cell receptors drive chronic lymphocytic leukemia by light‐chain‐dependent cross‐reaction with autoantigens
Source: EMBO Mol Med. 2017 Sep 12;9(11):1482–90. doi: 10.15252/emmm.201707732 (PMC5666309; doi:10.15252/emmm.201707732)
Supplement: Supplementary file 6 — Source Data for Expanded View [file EMMM-9-1482-s013.zip › EMM_07322_EV_SD/FigEV2/EMM_07322_FigEV2A_SD.pdf]

FIG EV2A

	VI10Yen x Eμ-TCL1					
**nAb titers**	320	320	640	640	640	320

	VI10Yen x Eμ-TCL1 + VSV							
**nAb titers**	163840	163840	81920	81920	81920	81920	163840	163840
