# Supplementary material for: Pathogen‐specific B‐cell receptors drive chronic lymphocytic leukemia by light‐chain‐dependent cross‐reaction with autoantigens
Source: EMBO Mol Med. 2017 Sep 12;9(11):1482–90. doi: 10.15252/emmm.201707732 (PMC5666309; doi:10.15252/emmm.201707732)
Supplement: Supplementary file 6 — Source Data for Expanded View [file EMMM-9-1482-s013.zip › EMM_07322_EV_SD/FigEV2/EMM_07322_FigEV2B_SD.pdf]

FIG EV2B

ORGANS	Eμ-TCL1								
SPLEEN	79.21	98.1	78.7	93.6	87.1	85.7	99.1	95.9	97.9
INGUINAL LNs	23.1	74.95	37.96	27.84	26.5	65.4	91.3	22.8	50.2
LIVER	85.06	98.11	82	93.8	94.1	96.7	99.6	91.2	98.2
PERITONEUM	94.11	94.94	92.61	96.7	67.9	94.8	99.2	93.4	96.8

ORGANS	Eμ-TCL1 + VSV						
SPLEEN	84.3	89.5	40.4	98.3	67.1	83.3	67.2
INGUINAL LNs	32.8	41.1	11.8	77.8	13.3	11.7	7.78
LIVER	80.5	89.6	27.7	98.8	68.1	85.5	55.5
PERITONEUM	63.6	98.1	53	92.8	96.3	92.3	93.9

ORGANS	VI10YEN x Eμ-TCL1							
SPLEEN	75.2	84.3	36.9	16	42.1	60.4	50.1	75.1
INGUINAL LNs	11.9	22.7	5.58	13.3	2.46	21.6	18.9	33
LIVER	86.8	91.5	36.9	18.5	37.6	44.9	41.3	57.9
PERITONEUM	65.6	91.5	83.6	2.61	10.7	7.35	13.2	70

ORGANS	VI10Yen x Eμ-TCL1 + VSV							
SPLEEN	6.92	63.1	91.4	91.7	70.4	6.74	74.8	56.4
INGUINAL LNs	8.44	48.4	9.71	43.1	7.45	7.48	48.6	14.3
LIVER	12.1	56.6	92	88.5	68.2	11	60	13.2
PERITONEUM	3.46	79.9	93	63.6	63.5	2.22	59.3	6.71
