# Supplementary material for: Pathogen‐specific B‐cell receptors drive chronic lymphocytic leukemia by light‐chain‐dependent cross‐reaction with autoantigens
Source: EMBO Mol Med. 2017 Sep 12;9(11):1482–90. doi: 10.15252/emmm.201707732 (PMC5666309; doi:10.15252/emmm.201707732)
Supplement: Supplementary file 6 — Source Data for Expanded View [file EMMM-9-1482-s013.zip › EMM_07322_EV_SD/FigEV3/EMM_07322_FigEV3_SD.pdf]

FIG EV3

Weeks	Eμ-TCL1																																																	
8										4.38	5.99	3.79	7.93						4.78	6.13	5.3	7.92																					1.77		1.6	2.61	2.38	2.51	2.7	2.17	3.1
12		4.69	8.95	5						7.7	14.6	3.21	9.43				7.63	8.68					18.4							7.97	2.64						1.66	1.47	1.65	2.51	1.03		2.65		2.84	7.1	5.41	4.37	7.08	2.94	5
16	13.3													6.36	8.47	16.8			7.62	27.6	6.18	12.6		9.04	11.3	6.46	7.33	7.55															3.37		2.39	11.8	13.7	4.82	18	2.16	5.15
20		12.3	26	6.96	15.3	24.5	46.3	6.09	8.32	18.8	42.3	24	24.5				45.5	16.9					39.5							34.5	4.5	19.3	20.3	16.8	8.97	12	4.52	9.98	2.55	4.06	4.74		5.98		9.31	17.9	24.6	8.94	30.3	3.2	7.41
24		23.3	32.6	21.9	36.6	49.9	58.3	11.5	27.9	38.5	34.5	58.5	43.7				46.6	25.3	35.2	78.6	6.79	30.9	42.9						12.2	45.9	9.49	36.7	26	23	14.2	17.9	20.2	29.2		24	9.68		12.9		17	21.6	37.6	20.7	16.4		24.4
28	54.3	53.4	43	21.5	19.7	33.2		20		23.7	37.4	50.2	21.7	45.2	58.8	68.5		28.8					54.7	83.3	92.6	16.9	14.6	34.7		52.2	9.32	75.6	50.8	26.9	33.4	23.8	24	36.4	12	23.3	19.6		16.8	5.36	36.8	56	62.3	30.5	26.8		35.7

Weeks	Eμ-TCL1 + LCMV															
8	6.97	4.97	6.22	7.9	6.41	7.78	5.7	4.5	6.64	5.02	6.24		6.55	8.47	4.14	8.08
12	3.95	3.13		3.27		3.27	2.62	5.51	4.17	2.1	2.67	2.71	2.21	2.08	2.93	
16	4.29	4.49		4.85		4.76	5.18	6.37	6.08	4.4	3.06	2.57	2.02	2.99	2.43	
20	3.77	3.66		3.71		2.73	4.73	5.83	4.41		2.73	2.87	2.18	2.19	3.06	
24	4.13		3.56		3.02	3.43	9.27	6.02		2.34	1.57	1.98	1.44	2.14	2.48	
28	3.37		3.29		2.62	4.04	7.85	7.36		2.53	1.67	2.38	1.57	2.84	2.31	

Weeks	KL25 x Eμ-TCL1																												
8	1.1	1.38	1.56	1.63	1.99	1.67	1.71			2.91																	1.56	1.63	1.38
12			3.64	1.25	5.01	2.19	2.18																				1.35	1.02	1.05
16								3.1	24.8	14	19.1	2.91	8.25														3.26	5.82	1.4
20	8.48	18.3	31.6	13.9	34.9	5.86	17.7	11.6	59.2	56.2	57.9	9.24	29.2	9.75	32.7	6.4	3.06	18	11.9	9.83	2.33	26.8	7.53	2.69	14.5	10.9	4.27	17.2	2.35
24	14.6		46	43.6	47.2	9.48	25.5							19.9	67.8	8.45	8.67	16	25.6	35.7	8.32	21.3	15.7	8.88	32.6	21.7	10	25.4	7.88
28		20.3	83	70	58.6	83.8	57.7	76	93	88.3	18.9	75.7											39.9						

Weeks	KL25 x Eμ-TCL1 + LCMV									
8	1.26	1.03	1.59	1.3	1.14	1.05	2.37	1.42	1.63	1.87
12	3.72	3	4.31	2.66	3.81	3.65	5.67	3.24		3.27
16	2.1	1.19	2.7	0.66	1.22	1.36	1.68	1.67		1.23
20	14.3	8.98	23.4	1.49	2.51	2.96	22	22		1.71
24	15.2	13.1	32	2.14	5.03	3.45	23.6	8.96		2.05
28	21.8	23.1	59.4	1.64	9.39	6.44	55.6	13.1		2.06
