# Supplementary material for: Pathogen‐specific B‐cell receptors drive chronic lymphocytic leukemia by light‐chain‐dependent cross‐reaction with autoantigens
Source: EMBO Mol Med. 2017 Sep 12;9(11):1482–90. doi: 10.15252/emmm.201707732 (PMC5666309; doi:10.15252/emmm.201707732)
Supplement: Supplementary file 6 — Source Data for Expanded View [file EMMM-9-1482-s013.zip › EMM_07322_EV_SD/FigEV4/EMM_07322_FigEV4A_SD.pdf]

FIG EV4A

Weeks	Eμ-TCL1					
**8**	1	1	1	1	1	1
**9**	1	1	1	1	1	1
**11**	1	1	1	1	1	1
**15**	1	1	1	1	1	1

Weeks	Eμ-TCL1 + LCMV-GP + Addavax					
**8**	1	1	1	1	1	1
**9**	1	1	1	1	1	1
**11**	1	1	1	1	1	1
**15**	1	1	1	1	1	1

Weeks	KL25 x Eμ-TCL1					
**8**	1	1	1	1	1	1
**9**	1	1	1	1	1	1
**11**	1	1	1	1	1	1
**15**	1	1	1	1	1	1

Weeks	KL25 x Eμ-TCL1 + LCMV-GP + Addavax										
**8**	1	1	1	1	1	1	1	1	1	1	1
**9**	1	1	1	1	1	1	1	1	1	1	1
**11**	1280	40	1280	113.1371	160	1810.193	1280	320	320	452.5	452.5483
**15**	1810.193	320	5120	5120	7240.8	10240	2560	1280	56.6	2560	7240.773
