# Supplementary material for: Pathogen‐specific B‐cell receptors drive chronic lymphocytic leukemia by light‐chain‐dependent cross‐reaction with autoantigens
Source: EMBO Mol Med. 2017 Sep 12;9(11):1482–90. doi: 10.15252/emmm.201707732 (PMC5666309; doi:10.15252/emmm.201707732)
Supplement: Supplementary file 6 — Source Data for Expanded View [file EMMM-9-1482-s013.zip › EMM_07322_EV_SD/FigEV4/EMM_07322_FigEV4B_SD.pdf]

FIG EV4B

Weeks	Eμ-TCL1															
8										4.38	5.99	3.79	7.93			
12		4.69	8.95	5						7.7	14.6	3.21	9.43			
16	13.3													6.36	8.47	16.8
20		12.3	26	6.96	15.3	24.5	46.3	6.09	8.32	18.8	42.3	24	24.5			
24		23.3	32.6	21.9	36.6	49.9	58.3	11.5	27.9	38.5	34.5	58.5	43.7			
28	54.3	53.4	43	21.5	19.7	33.2		20		23.7	37.4	50.2	21.7	45.2	58.8	68.5
32	93.2	52.2	10.8	74	36.8	19.5	31	39.9	21.6	59.2	13.3			43.8	77.4	

Weeks	Eμ-TCL1 (cont.)																
8			4.78	6.13	5.3	7.92											
12	7.63	8.68					18.4							7.97	2.64		
16			7.62	27.6	6.18	12.6		9.04	11.3	6.46	7.33	7.55					
20	45.5	16.9					39.5							34.5	4.5	19.3	20.3
24	46.6	25.3	35.2	78.6	6.79	30.9	42.9						12.2	45.9	9.49	36.7	26
28		28.8					54.7	83.3	92.6	16.9	14.6	34.7		52.2	9.32	75.6	50.8
32	79.9	34	65	79.1	14.5	75.2	18	72.6	7.72	41	60.9	67.6		77.6	8.74		85.2

Weeks	Eμ-TCL1 (cont.)																
8										1.77		1.6	2.61	2.38	2.51	2.7	2.17	3.1
12				1.66	1.47	1.65	2.51	1.03		2.65		2.84	7.1	5.41	4.37	7.08	2.94	5
16										3.37		2.39	11.8	13.7	4.82	18	2.16	5.15
20	16.8	8.97	12	4.52	9.98	2.55	4.06	4.74		5.98		9.31	17.9	24.6	8.94	30.3	3.2	7.41
24	23	14.2	17.9	20.2	29.2		24	9.68		12.9		17	21.6	37.6	20.7	16.4		24.4
28	26.9	33.4	23.8	24	36.4	12	23.3	19.6		16.8	5.36	36.8	56	62.3	30.5	26.8		35.7
32	60.4	59.7	30.8	42.7	40	26.8	54.5	15.7		22.8	13.9	48.5	45.1	60.8	30.5	55.5	35.3	24.9

Weeks	Eμ-TCL1 + Addavax					
8	4.88	5.2	4.96	6.01	5.03	5.09
12	6.36	9.95	5.74	5.55	4.56	4.65
16	11.4	15.5	8.95	8.55	7.61	10.2
20	18.3	19.8	11.6	9.24	9.04	49
24	37.8	23.2	71.4	19.2	19.8	77.5
28	64.2	2.55	94.8	17.4	37.7	77.1
32	70.3	2.46	96.5	30.4	49.8	36.7

Weeks	Eμ-TCL1 + LCMV-GP + Addavax					
8	6.48	6.8	4.42	7.8	4	8.27
12	8.98	9.33	5.11	13.6	5.58	13.6
16	14.8	16.8	11.3	24.2	10.3	23.5
20	19.5	26.5	12.8	20	13.2	23.8
24	34.3	67.6	16.2	29.7	23.3	32
28	65.8	92.9	34.2	39.9	41.4	59.8
32	64.9	96	61.4	48.5	42.7	
