# Supplementary material for: Pathogen‐specific B‐cell receptors drive chronic lymphocytic leukemia by light‐chain‐dependent cross‐reaction with autoantigens
Source: EMBO Mol Med. 2017 Sep 12;9(11):1482–90. doi: 10.15252/emmm.201707732 (PMC5666309; doi:10.15252/emmm.201707732)
Supplement: Supplementary file 6 — Source Data for Expanded View [file EMMM-9-1482-s013.zip › EMM_07322_EV_SD/FigEV4/EMM_07322_FigEV4C_SD.pdf]

FIG EV4C

ORGANS	Eμ-TCL1							
SPLEEN	79.21	98.1	78.7	93.6	87.1	85.7	99.1	95.9	97.9
INGUINAL LNs	23.1	74.95	37.96	27.84	26.5	65.4	91.3	22.8	50.2
LIVER	85.06	98.11	82	93.8	94.1	96.7	99.6	91.2	98.2
PERITONEUM	94.11	94.94	92.61	96.7	67.9	94.8	99.2	93.4	96.8

ORGANS	Eμ-TCL1 + LCMV-GP + Addavax				
SPLEEN	77.8	95.3	78.9	81.1	80.5
INGUINAL LNs	13.4	87.9	23.2	34.9	29.7
LIVER	84.6	97.2	91	82.9	85
PERITONEUM	97.8	84.5	58.7	98.1	88.2

ORGANS	KL25 x Eμ-TCL1					
SPLEEN	53.1	95.1	84.6	88.5	49.1	87.6
INGUINAL LNs	41.5	31.3	22.26	50.31	60.7	90.6
LIVER	60.9	93.9	82.71	81.87	78.2	91.5
PERITONEUM	92.96	89.9	63.33	96.5	74.2	92.2

ORGANS	KL25 x Eμ-TCL1 + LCMV-GP + Addavax						
SPLEEN	40	75.2	46.2	82.8	86.1	68	81.8
INGUINAL LNs	23	88.2	26.9	56.2	29.1	15.5	71.6
LIVER	63.3	83.3	89.8	86.5	59.1	99.8	63.8
PERITONEUM	95.8	59.3	94.9	79.9	92.6	90.9	90.6
