# Supplementary material for: Pathogen‐specific B‐cell receptors drive chronic lymphocytic leukemia by light‐chain‐dependent cross‐reaction with autoantigens
Source: EMBO Mol Med. 2017 Sep 12;9(11):1482–90. doi: 10.15252/emmm.201707732 (PMC5666309; doi:10.15252/emmm.201707732)
Supplement: Supplementary file 8 — Source Data for Figure 1A [file EMMM-9-1482-s006.pdf]

FIG 1A

Weeks	WT																			
8	1.16	1.15	0.915	0.976																
12	1.63	1.66	1.18	1.53	1.6	1.66														
16	0.893	1.24	1.02	0.942	0.726	1.37	1.17	1.1	1.21	1.78	4.67	3.42	0.585	2.78	0.936	6.46	3.03	0.724	0.791	0.934
20	1.55	1.33	1.24	1.4	1.22	1.59	1.8	1.4	1.78	1.83	1.93	1.26	1.87	1.93	1.29	2	1.83	1.24	1.05	1.82
24	1.01	1.02	0.899	0.94	1.25	1.36	1.54	1.54	1.66	1.65	1.57	1.36	1.39	1.2	1.3	1.14	1.16	1.04	0.677	1.16
28	0.541	0.499	0.606	0.601	0.617	0.777	0.821	0.985	0.83	0.911	0.779		0.705	0.579	0.811	0.727	0.81	0.676	0.443	0.631
32	0.548		0.706	0.645	1.85	1.03	1.06	0.857	0.955	0.88	0.652		0.904	0.547	1.04	0.606	0.963	0.666	0.456	0.871
36	0.573		0.573	0.979	3.13	1.16	1.06	0.877	1	1.24	0.673		0.831	0.823	1.02	0.741	0.837	1.43	0.544	0.655

Weeks	Eμ-TCL1																																																	
8										4.38	5.99	3.79	7.93						4.78	6.13	5.3	7.92																				1.77		1.6	2.61	2.38	2.51	2.7	2.17	3.1
12		4.69	8.95	5						7.7	14.6	3.21	9.43				7.63	8.68					18.4							7.97	2.64						1.66	1.47	1.65	2.51	1.03	2.65		2.84	7.1	5.41	4.37	7.08	2.94	5
16	13.3													6.36	8.47	16.8			7.62	27.6	6.18	12.6		9.04	11.3	6.46	7.33	7.55														3.37		2.39	11.8	13.7	4.82	18	2.16	5.15
20		12.3	26	6.96	15.3	24.5	46.3	6.09	8.32	18.8	42.3	24	24.5				45.5	16.9					39.5							34.5	4.5	19.3	20.3	16.8	8.97	12	4.52	9.98	2.55	4.06	4.74	5.98		9.31	17.9	24.6	8.94	30.3	3.2	7.41
24		23.3	32.6	21.9	36.6	49.9	58.3	11.5	27.9	38.5	34.5	58.5	43.7				46.6	25.3	35.2	78.6	6.79	30.9	42.9						12.2	45.9	9.49	36.7	26	23	14.2	17.9	20.2	29.2		24	9.68	12.9		17	21.6	37.6	20.7	16.4		24.4
28	54.3	53.4	43	21.5	19.7	33.2		20		23.7	37.4	50.2	21.7	45.2	58.8	68.5		28.8					54.7	83.3	92.6	16.9	14.6	34.7		52.2	9.32	75.6	50.8	26.9	33.4	23.8	24	36.4	12	23.3	19.6	16.8	5.36	36.8	56	62.3	30.5	26.8		35.7
32	93.2	52.2	10.8	74	36.8	19.5	31	39.9	21.6	59.2	13.3			43.8	77.4		79.9	34	65	79.1	14.5	75.2	18	72.6	7.72	41	60.9	67.6		77.6	8.74		85.2	60.4	59.7	30.8	42.7	40	26.8	54.5	15.7	22.8	13.9	48.5	45.1	60.8	30.5	55.5	35.3	24.9
36	46	50.1	85.3	40.3	79.4	70.6	22.7	24.3	92.6		83.3				82.1			33.1			59.7		73.4							85.9	37.7	42.5	43.9	25.7	69.5	22.9	30.9	45.8	38.2	67.6	22.1							61.8		32.8

Weeks	KL25 x Eμ-TCL1																													
8	1.1	1.38	1.56	1.63	1.99	1.67	1.71			2.91																	1.56	1.63	1.38	
12			3.64	1.25	5.01	2.19	2.18																				1.35	1.02	1.05	
16								3.1	24.8	14	19.1	2.91	8.25														3.26	5.82	1.4	
20	8.48	18.3	31.6	13.9	34.9	5.86	17.7	11.6	59.2	56.2	57.9	9.24	29.2	9.75	32.7	6.4	3.06	18	11.9	9.83	2.33	26.8	7.53	2.69	14.5	10.9	4.27	17.2	2.35	
24	14.6		46	43.6	47.2	9.48	25.5							19.9	67.8	8.45	8.67	16	25.6	35.7	8.32	21.3	15.7	8.88	32.6	21.7	10	25.4	7.88	
28		20.3	83	70	58.6	83.8	57.7	76	93	88.3	18.9	75.7											39.9							
32	52.7	44.4	94.1	8.74	37.1	92		94.4	94		31.5	73.4	17.3	69	78.5	6.95	28.8	10.1	61.7	43.6										
36	63.7					95.6		60.3	22.1		89.2			85.2		46.5	93.4	22.1	96.6	92.6	17.3									

Weeks	Vl10Yen x Eμ-TCL1																																							
8	2.63	1.96	1.69	1.23	2.18	1.47	1.71	1.28	1.05	4.78	4.64	4.54	4.47																											
12	1.01	0.871	1.03	0.709	1.1	0.651	0.509	0.804	0.439	0.947	0.976	0.629	1.51							0.753	0.916	1.01										1.03	1.95		1.56	4.28	2.85	4.76	2.92	2.65	2.45
16	1.11	1.29								1.17	0.598	2.72	3.57	0.972	0.873		0.936	7.17	0.59																					
20	3.23	2.02	0.922	0.58	8.07		0.655	1.52	0.625	1.96	1.26	0.634	8.66							1.3	2.13	1.42																		
24	7.01	2.41	0.998	0.623	4.72		1.07	12.1	0.559	9.21	1.42	0.505	20.3	6.16	0.503		1.25	18.3	1.54	7.56	7.75	7.63																		
28	37.5	29.8	1.77	0.964	11.5	16.6	17.7	22.8	1.42	51.8	2.03	2.48	20.8	11.6	0.512	3.07	1.73	32.2	1.23	28.9	10.7	14.1	11	1.53	1.24	16.3	14.5	6.72	8.91											
32	58.2	51.4	4.39	2.9	45.8	1.18	9.44	27.3	5.51	72	2.52	2	25.2	50.2	51.1	2.47	5.4	30.5	3.85	30.8	12.1	35.8	73.4	4.26	2.38	30.4	10.3	0.788		2.35	44.7	2.34	40.2	12.7	28.4					
36	90.2	80.3	10.1	3.01	67.3	74.2	13.6	52.4	26.3	79.1	0.987	3.8	41.6	29.9	7.94	1.75	8.57	72.4	12.3	39.8	5.08	73.1	3.91	5.67	38.4	26.8	1.76		3.25	51	6.15	48.1	24.7	76.2						

Weeks	D_HLMP2A x Eμ-TCL1																																															
8																			2.51	3.49	3.43	3.18	4.4	4.3	4.15	3.72	5.44	4.97	3.72	4.49	2.62	4.57	3.56	5.06	4.78	4.12												
12	1.3		2.42	2.61	2.77	3.56	2.46	2.73	5	4.64	5.58	3.65	6.05	3	4.07	3.86	4.67	3.39			3.66	6.05	8.14	3.7	6.32		3.45	3.64	3.72	1.94	2.82	3.5	2.69	2.95	2.87	3.65												
16	2.62	3.47	1.66	2.04	1.98	2.2	2.28	1.72	2.93	3.85	3.11	3.12	3.53	3.95	1.94	1.86	2.14	2.24	2.5	3.77	2.81	1.79	2.97	3.88	3.55	1.29	1.84	1.55				5.33	4.92	2.96														
20	1.81	1.88	1.86	1.63	1.9	1.62	1.37		2.42	2.73	2.15	3.26	1.31	0.947	1.44	1.14	1.43	1.2	0.94	0.829	2.04	1.24	2.67	1.28	1.87							5.32	8.9	4.4														
24	0.66	1.15	1.29	0.483	1	2.29	0.51		2.9	1.9	1.91	3.45	0.946	0.972	1.37	0.771	1.33	1.12																12.9														
28	3.04	3.86	2.62	3.07	3.73	10	2.49		4.89	5.51	2.99	3.96	1.17	1.09		3.8		2.36	3.83	1.56	3.75	3.48	25.5	4.48	2.27	1.53	3.33	1.63	1.99	2.49	5.86	2.15	1.51	3.11	1.94	1.68	1.38	30.2	2.22	1.66	3.88	3.27	2.66	4.88	0.98	0.99		2.28
32	1.66	6.22	2.54	2.69	3.67	11	1.25		8.13	4.4	4.7	6.04		1.16		2.36		2.32	0.917	1.76	1.78	1.73	23.9	2.95	2.14	2.41	2.73	2.92	2.6	23.3	5.55	4.94																
36	4.3	17.4	4.21	3.05	2.91	35.8	3.32	12.1	11.5	10.1	8.44	7.45	1.07	5.83	1.89	1.5	1.98	2.87	2.33	3.42	6.69	4.36	33.3	6.58	9.57	1.51	11.5	4.3	2.31	4.5		0.795				2.32	3.61	4.3	11.4	4.09	41.1	8.44	6.28					
