# Supplementary material for: Pathogen‐specific B‐cell receptors drive chronic lymphocytic leukemia by light‐chain‐dependent cross‐reaction with autoantigens
Source: EMBO Mol Med. 2017 Sep 12;9(11):1482–90. doi: 10.15252/emmm.201707732 (PMC5666309; doi:10.15252/emmm.201707732)
Supplement: Supplementary file 9 — Source Data for Figure 1B [file EMMM-9-1482-s007.pdf]

FIG 1B

Weeks	WT	Eμ-TCL1	KL25 x Eμ-TCL1	VI10Yen x Eμ-TCL1	DHLMP2A x Eμ-TCL1
36	0				
36	0				
36	0				
36	0				
36	0				
36	0				
36	0				
36	0				
36	0				
36	0				
36	0				
36	0				
36	0				
36	0				
36	0				
36	0				
36	0				
36	0				
36	0				
36	0				
22		1			
24		1			
21		1			
24		1			
22		1			
20		1			
20		1			
25		1			
24		1			
19		1			
19		1			
27		1			
22		1			
18		1			
24		1			
19		1			
16		1			
36		1			
30		1			
25		1			
14		1			
22		1			
18		1			
22		1			
30		1			
24		1			
34		1			
15		1			
36		1			
20		1			
20		1			
22		1			
22		1			
22		1			
25		1			
25		1			
30		1			
27		1			
35		1			
27		1			
35		1			
27		1			
22		1			
20		1			
25		1			
18		1			
25		1			
31			1		
21			1		
19			1		
24			1		
19			1		
27			1		
24			1		
27			1		
18			1		
18			1		
21			1		
31			1		
36			1		
31			1		
35			1		
25			1		
25			1		
25			1		
36			0		
20			1		
28			1		
31			0		
25			1		
25			1		
25			1		
29				1	
29				1	
36				0	
36				0	
30				1	
36				0	
36				0	
36				1	
29				1	
36				0	
35				0	
29				1	
34				1	
36				0	
34				0	
25				1	
36				0	
29				1	
34				1	
31				1	
36					0
36					0
36					0
36					0
36					0
36					1
36					0
36					0
36					0
36					0
29					1
36					0
29					0
34					0
32					0
36					0
36					0
36					0
36					0
36					0
36					0
36					0
26					1
