# Supplementary material for: Pathogen‐specific B‐cell receptors drive chronic lymphocytic leukemia by light‐chain‐dependent cross‐reaction with autoantigens
Source: EMBO Mol Med. 2017 Sep 12;9(11):1482–90. doi: 10.15252/emmm.201707732 (PMC5666309; doi:10.15252/emmm.201707732)
Supplement: Supplementary file 10 — Source Data for Figure 1C [file EMMM-9-1482-s008.pdf]

FIG 1C

ORGANS	WT					
SPLEEN	4.87	6.54	4.34	2.89	5.47	7.77
INGUINAL LNs	4.96	4.94	6.47	3.92	3.7	5.33
LIVER	1.93	2.74	2.38	2.11	1.51	1.85
PERITONEUM	16	10.3	20	10.8	19	17.8

ORGANS	Eμ-TCL1													
SPLEEN	37.6	64.9	50.8	63.9	83.14	79.21	98.1	78.7	93.6	87.1	85.7	99.1	95.9	97.9
INGUINAL LNs	13.34	16.98	12.1	19.54	16.7	23.1	74.95	37.96	27.84	26.5	65.4	91.3	22.8	50.2
LIVER	40.3	72.31	26.4	65.79	89.07	85.06	98.11	82	93.8	94.1	96.7	99.6	91.2	98.2
PERITONEUM	88.76	93.04	75.31	97.1	93.4	94.11	94.94	92.61	96.7	67.9	94.8	99.2	93.4	96.8

ORGANS	KL25 x Eμ-TCL1												
SPLEEN	68.6	69.9	82.09	52.3	65.74	53.1	58.58	54.9	95.1	84.6	88.5	49.1	87.6
INGUINAL LNs	17.7	20.6	19.1	7.6	18.5	41.5	31.5	20	31.3	22.26	50.31	60.7	90.6
LIVER	25.7	39.5	60.3	41.5	55.33	60.9	46.73	62.4	93.9	82.71	81.87	78.2	91.5
PERITONEUM	86.92	49.94	77.36	78.59	93.111	92.96	62.88	77.78	89.9	63.33	96.5	74.2	92.2

ORGANS	Vl10Yen x Eμ-TCL1							
SPLEEN	75.2	84.3	36.9	16	42.1	60.4	50.1	75.1
INGUINAL LNs	11.9	22.7	5.58	13.3	2.46	21.6	18.9	33
LIVER	86.8	91.5	36.9	18.5	37.6	44.9	41.3	57.9
PERITONEUM	65.6	91.5	83.6	2.61	10.7	7.35	13.2	70

ORGANS	D_HLMP2A x Eμ-TCL1											
SPLEEN	50.9	68.2	20	87.6	59.8	30.9	9.92	18.4	84	18.7	9.38	12.9
INGUINAL LNs	13	41.3	6.72	80.6	12.3	36.9	5.55	13.2	21.6	9.93	3.66	4.51
LIVER	41	46.5	10.8	91	42.3	12.9	10.7	10.7	76.9	12.3	3.65	3.68
PERITONEUM	4.59	45.1	1.33	95.2	4.92	3.83	1.77	2.09	64.8	1.44	0.716	2.68
