# Supplementary material for: Pathogen‐specific B‐cell receptors drive chronic lymphocytic leukemia by light‐chain‐dependent cross‐reaction with autoantigens
Source: EMBO Mol Med. 2017 Sep 12;9(11):1482–90. doi: 10.15252/emmm.201707732 (PMC5666309; doi:10.15252/emmm.201707732)
Supplement: Supplementary file 11 — Source Data for Figure 2A [file EMMM-9-1482-s009.pdf]

FIG 2A

Weeks	Eμ-TCL1												
8										4.38	5.99	3.79	7.93
12		4.69	8.95	5						7.7	14.6	3.21	9.43
16	13.3												
20		12.3	26	6.96	15.3	24.5	46.3	6.09	8.32	18.8	42.3	24	24.5
24		23.3	32.6	21.9	36.6	49.9	58.3	11.5	27.9	38.5	34.5	58.5	43.7
28	54.3	53.4	43	21.5	19.7	33.2		20		23.7	37.4	50.2	21.7
32	93.2	52.2	10.8	74	36.8	19.5	31	39.9	21.6	59.2	13.3		
36	46	50.1	85.3	40.3	79.4	70.6	22.7	24.3	92.6		83.3		

Weeks													
8						4.78	6.13	5.3	7.92				
12				7.63	8.68					18.4			
16	6.36	8.47	16.8			7.62	27.6	6.18	12.6		9.04	11.3	6.46
20				45.5	16.9					39.5			
24				46.6	25.3	35.2	78.6	6.79	30.9	42.9			
28	45.2	58.8	68.5		28.8					54.7	83.3	92.6	16.9
32	43.8	77.4		79.9	34	65	79.1	14.5	75.2	18	72.6	7.72	41
36		82.1			33.1			59.7		73.4			

Weeks													
8													
12				7.97	2.64						1.66	1.47	1.65
16	7.33	7.55											
20				34.5	4.5	19.3	20.3	16.8	8.97	12	4.52	9.98	2.55
24			12.2	45.9	9.49	36.7	26	23	14.2	17.9	20.2	29.2	
28	14.6	34.7		52.2	9.32	75.6	50.8	26.9	33.4	23.8	24	36.4	12
32	60.9	67.6		77.6	8.74		85.2	60.4	59.7	30.8	42.7	40	26.8
36				85.9	37.7	42.5	43.9	25.7	69.5	22.9	30.9	45.8	38.2

Weeks												
8				1.77		1.6	2.61	2.38	2.51	2.7	2.17	3.1
12	2.51	1.03		2.65		2.84	7.1	5.41	4.37	7.08	2.94	5
16				3.37		2.39	11.8	13.7	4.82	18	2.16	5.15
20	4.06	4.74		5.98		9.31	17.9	24.6	8.94	30.3	3.2	7.41
24	24	9.68		12.9		17	21.6	37.6	20.7	16.4		24.4
28	23.3	19.6		16.8	5.36	36.8	56	62.3	30.5	26.8		35.7
32	54.5	15.7		22.8	13.9	48.5	45.1	60.8	30.5	55.5	35.3	24.9
36	67.6	22.1								61.8		32.8

Weeks	Vl10Yen x Eμ-TCL1														
8	2.63	1.96	1.69	1.23	2.18	1.47	1.71	1.28	1.05	4.78	4.64	4.54	4.47		
12	1.01	0.871	1.03	0.709	1.1	0.651	0.509	0.804	0.439	0.947	0.976	0.629	1.51		
16	1.11	1.29								1.17	0.598	2.72	3.57	0.972	0.873
20	3.23	2.02	0.922	0.58	8.07		0.655	1.52	0.625	1.96	1.26	0.634	8.66		
24	7.01	2.41	0.998	0.623	4.72		1.07	12.1	0.559	9.21	1.42	0.505	20.3	6.16	0.503
28	37.5	29.8	1.77	0.964	11.5	16.6	17.7	22.8	1.42	51.8	2.03	2.48	20.8	11.6	0.512
32	58.2	51.4	4.39	2.9	45.8	1.18	9.44	27.3	5.51	72	2.52	2	25.2	50.2	51.1
36	90.2	80.3	10.1	3.01	67.3	74.2	13.6	52.4	26.3	79.1	0.987	3.8	41.6	29.9	7.94

Weeks														
8														
12					0.753	0.916	1.01							
16		0.936	7.17	0.59										
20					1.3	2.13	1.42							
24		1.25	18.3	1.54	7.56	7.75	7.63							
28	3.07	1.73	32.2	1.23	28.9	10.7	14.1	11	1.53	1.24	16.3	14.5	6.72	8.91
32	2.47	5.4	30.5	3.85	30.8	12.1	35.8	73.4	4.26	2.38	30.4	10.3	0.788	
36	1.75	8.57	72.4	12.3	39.8	5.08	73.1	3.91	5.67	38.4	26.8	1.76		3.25

Weeks														
8														
12			1.03	1.95		1.56	4.28	2.85	4.76	2.92	2.65	2.45		
16														
20														
24														
28														
32	2.35	44.7	2.34	40.2	12.7	28.4							8.44	6.28
36	51	6.15	48.1	24.7	76.2									

Weeks	Eμ-TCL1 + VSV										
8	1.79	1.69	1.7	0.369	4.17	1.08	3.03	2.35			
12	5.14	5.37	3.26	6.48	6		2.28	4.15	2.92	3.17	
16	6.5	9	4.73	20.6	10.7		3.61	5.28	2.37	2.98	
20		12.6	29.8	5.58	41	29.4		8.63	9.82	4.12	8.3
24		20.2	39.1	9.61	48	58.8		10.7	13.2	5.43	15
28	26.6	44.4	8.75	80.6	70		14	22.6	7.62	16	
32	34.2	59.3	11.4	90.7	40.7		18.9	84.8	30	18.7	
36	63	85.8	13.9	97.1			42.3		77.1	63.8	

Weeks	Vl10Yen x Eμ-TCL1 + VSV									
8	1.29	1.38	1.4	1.97	1.43	1.07	0.901		0.956	
12	2.64	6.34	3.52	4.96	3.86	1.64	1.37		1.92	
16	2.64	5.29	16.2	5.83	5.35	1.45	2.48		1.92	
20	21	2.19	7.54		19.5	10	1.87	4.51		2.78
24	25	1.52	8.14		32.7	8.44	1.74	6.45		2.02
28	0.488	7.43		43	10.9	0.668	8.8		0.909	
32	2.2	16.7		40.4	13.6	1.49	38.8		2.02	
36	3.2	36.5		86	39.2	7.15	88.7		12.3	
