# Supplementary material for: Pathogen‐specific B‐cell receptors drive chronic lymphocytic leukemia by light‐chain‐dependent cross‐reaction with autoantigens
Source: EMBO Mol Med. 2017 Sep 12;9(11):1482–90. doi: 10.15252/emmm.201707732 (PMC5666309; doi:10.15252/emmm.201707732)
Supplement: Supplementary file 12 — Source Data for Figure 2B [file EMMM-9-1482-s010.pdf]

FIG 2B

Weeks	Eμ-TCL1	Eμ-TCL1 + VSV	Vl10Yen x Eμ-TCL1	Vi10YEN x Eμ-TCL1 + VSV
22	1			
24	1			
21	1			
24	1			
22	1			
20	1			
20	1			
25	1			
24	1			
19	1			
19	1			
27	1			
22	1			
18	1			
24	1			
19	1			
16	1			
36	1			
30	1			
25	1			
14	1			
22	1			
18	1			
22	1			
30	1			
24	1			
34	1			
15	1			
36	1			
20	1			
20	1			
22	1			
22	1			
22	1			
25	1			
25	1			
30	1			
27	1			
35	1			
27	1			
35	1			
27	1			
22	1			
20	1			
25	1			
18	1			
25	1			
29			1	
29			1	
36			0	
36			0	
30			1	
36			0	
36			0	
36			1	
29			1	
36			0	
35			0	
29			1	
34			1	
36			0	
34			0	
25			1	
36			0	
29			1	
34			1	
31			1	
35				0
32				1
19				1
25				1
29				1
35				0
29				1
36				0
25		1		
19		1		
36		0		
17		1		
21		1		
32		1		
27		1		
29		1		
33		1		
