# Supplementary material for: Pathogen‐specific B‐cell receptors drive chronic lymphocytic leukemia by light‐chain‐dependent cross‐reaction with autoantigens
Source: EMBO Mol Med. 2017 Sep 12;9(11):1482–90. doi: 10.15252/emmm.201707732 (PMC5666309; doi:10.15252/emmm.201707732)
Supplement: Supplementary file 13 — Source Data for Figure 2C [file EMMM-9-1482-s011.pdf]

FIG 2C

Weeks	Eμ-TCL1																																																		
**8**										4.38	5.99	3.79	7.93						4.78	6.13	5.3	7.92																					1.77		1.6	2.61	2.38	2.51	2.7	2.17	3.1
**12**		4.69	8.95	5						7.7	14.6	3.21	9.43				7.63	8.68					18.4							7.97	2.64						1.66	1.47	1.65	2.51	1.03		2.65		2.84	7.1	5.41	4.37	7.08	2.94	5
**16**	13.3													6.36	8.47	16.8			7.62	27.6	6.18	12.6		9.04	11.3	6.46	7.33	7.55															3.37		2.39	11.8	13.7	4.82	18	2.16	5.15
**20**		12.3	26	6.96	15.3	24.5	46.3	6.09	8.32	18.8	42.3	24	24.5				45.5	16.9					39.5							34.5	4.5	19.3	20.3	16.8	8.97	12	4.52	9.98	2.55	4.06	4.74		5.98		9.31	17.9	24.6	8.94	30.3	3.2	7.41
**24**		23.3	32.6	21.9	36.6	49.9	58.3	11.5	27.9	38.5	34.5	58.5	43.7				46.6	25.3	35.2	78.6	6.79	30.9	42.9						12.2	45.9	9.49	36.7	26	23	14.2	17.9	20.2	29.2		24	9.68		12.9		17	21.6	37.6	20.7	16.4		24.4
**28**	54.3	53.4	43	21.5	19.7	33.2		20		23.7	37.4	50.2	21.7	45.2	58.8	68.5		28.8					54.7	83.3	92.6	16.9	14.6	34.7		52.2	9.32	75.6	50.8	26.9	33.4	23.8	24	36.4	12	23.3	19.6		16.8	5.36	36.8	56	62.3	30.5	26.8		35.7
**32**	93.2	52.2	10.8	74	36.8	19.5	31	39.9	21.6	59.2	13.3			43.8	77.4		79.9	34	65	79.1	14.5	75.2	18	72.6	7.72	41	60.9	67.6		77.6	8.74		85.2	60.4	59.7	30.8	42.7	40	26.8	54.5	15.7		22.8	13.9	48.5	45.1	60.8	30.5	55.5	35.3	24.9

Weeks	Eμ-TCL1 + LCMV-GP + Addavax					
**8**	6.48	6.8	4.42	7.8	4	8.27
**12**	8.98	9.33	5.11	13.6	5.58	13.6
**16**	14.8	16.8	11.3	24.2	10.3	23.5
**20**	19.5	26.5	12.8	20	13.2	23.8
**24**	34.3	67.6	16.2	29.7	23.3	32
**28**	65.8	92.9	34.2	39.9	41.4	59.8
**32**	64.9	96	61.4	48.5	42.7	

Weeks	KL25 x Eμ-TCL1																												
**8**	1.1	1.38	1.56	1.63	1.99	1.67	1.71			2.91																	1.56	1.63	1.38
**12**			3.64	1.25	5.01	2.19	2.18																				1.35	1.02	1.05
**16**								3.1	24.8	14	19.1	2.91	8.25														3.26	5.82	1.4
**20**	8.48	18.3	31.6	13.9	34.9	5.86	17.7	11.6	59.2	56.2	57.9	9.24	29.2	9.75	32.7	6.4	3.06	18	11.9	9.83	2.33	26.8	7.53	2.69	14.5	10.9	4.27	17.2	2.35
**24**	14.6		46	43.6	47.2	9.48	25.5							19.9	67.8	8.45	8.67	16	25.6	35.7	8.32	21.3	15.7	8.88	32.6	21.7	10	25.4	7.88
**28**		20.3	83	70	58.6	83.8	57.7	76	93	88.3	18.9	75.7											39.9						
**32**	52.7	44.4	94.1	8.74	37.1	92		94.4	94		31.5	73.4	17.3	69	78.5	6.95	28.8	10.1	61.7	43.6									

Weeks	KL25 x Eμ-TCL1 + LCMV-GP + Addavax										
**8**	1.65	6.36	0.809	1.45	1.76	1.35	0.675	1.37	1.1	1.2	1.37
**12**	3.39	17.4	0.905	6.83	2.17	1.21	1.01	19.4	2.27	6.82	2.8
**16**	18.5	45.3	6.48	37.1	14.6	15.6	5.68	55.9	10.2	30.3	28.1
**20**	19.5	41.3	9.73	54	16.7	23.5	3.9	50.5	12.2	32.1	21.4
**24**	42.6	83.1	22.8	91.2	28.7	37.5	7.55	56.6	22.2	61.7	9.48
**28**	26.4	94.3	32.9	89.7	53.4	30	10.2	69	26.7	71.9	4.23
**32**	29	65.4	56.5	82.3	72.8	44.8	8.3	31.1	42	70.5	
