# Supplementary material for: Pathogen‐specific B‐cell receptors drive chronic lymphocytic leukemia by light‐chain‐dependent cross‐reaction with autoantigens
Source: EMBO Mol Med. 2017 Sep 12;9(11):1482–90. doi: 10.15252/emmm.201707732 (PMC5666309; doi:10.15252/emmm.201707732)
Supplement: Supplementary file 14 — Source Data for Figure 2D [file EMMM-9-1482-s012.pdf]

FIG 2D

Weeks	Eμ-TCL1	Eμ-TCL1 + LCMV-GP + Addavax	KL25 x Eμ-TCL1	KL25 x Eμ-TCL1 + LCMV-GP + Addavax
22	1			
24	1			
21	1			
24	1			
22	1			
20	1			
20	1			
25	1			
24	1			
19	1			
19	1			
27	1			
22	1			
18	1			
24	1			
19	1			
16	1			
32	0			
30	1			
25	1			
14	1			
22	1			
18	1			
22	1			
30	1			
24	1			
32	0			
15	1			
32	0			
20	1			
20	1			
22	1			
22	1			
22	1			
25	1			
25	1			
30	1			
27	1			
32	0			
27	1			
32	0			
27	1			
22	1			
20	1			
25	1			
18	1			
25	1			
20		1		
20		1		
28		1		
16		1		
24		1		
32		1		
16		1		
31			1	
21			1	
19			1	
24			1	
19			1	
27			1	
24			1	
27			1	
16			1	
18			1	
18			1	
27			1	
21			1	
31			1	
20			1	
32			0	
25			1	
25			1	
32			0	
20			1	
28			1	
25			1	
25			1	
25			1	
24				1
16				1
24				1
16				1
24				1
20				1
32				0
16				1
24				1
16				1
16				1
